# Comparative transcriptome analysis to investigate the high starch accumulation of duckweed (*Landoltia punctata*) under nutrient starvation

**DOI:** 10.1186/1754-6834-6-72

**Published:** 2013-05-08

**Authors:** Xiang Tao, Yang Fang, Yao Xiao, Yan-ling Jin, Xin-rong Ma, Yun Zhao, Kai-ze He, Hai Zhao, Hai-yan Wang

**Affiliations:** 1 Chengdu Institute of Biology, Chinese Academy of Sciences, Chengdu, Sichuan, 610041, China; 2 College of Life Sciences, Key Laboratory of Bio-resources and Eco-environment, Ministry of Education, Sichuan Key Laboratory of Molecular Biology and Biotechnology, Sichuan University, Chengdu, Sichuan, 610064, China

**Keywords:** Duckweed, Transcriptome, Bioethanol, Nutrient starvation, Starch accumulation, Metabolic flux

## Abstract

**Background:**

Duckweed can thrive on anthropogenic wastewater and produce tremendous biomass production. Due to its relatively high starch and low lignin percentage, duckweed is a good candidate for bioethanol fermentation. Previous studies have observed that water devoid of nutrients is good for starch accumulation, but its molecular mechanism remains unrevealed.

**Results:**

This study globally analyzed the response to nutrient starvation in order to investigate the starch accumulation in duckweed (*Landoltia punctata*). *L. punctata* was transferred from nutrient-rich solution to distilled water and sampled at different time points. Physiological measurements demonstrated that the activity of ADP-glucose pyrophosphorylase, the key enzyme of starch synthesis, as well as the starch percentage in duckweed, increased continuously under nutrient starvation. Samples collected at 0 h, 2 h and 24 h time points respectively were used for comparative gene expression analysis using RNA-Seq. A comprehensive transcriptome, comprising of 74,797 contigs, was constructed by a de novo assembly of the RNA-Seq reads. Gene expression profiling results showed that the expression of some transcripts encoding key enzymes involved in starch biosynthesis was up-regulated, while the expression of transcripts encoding enzymes involved in starch consumption were down-regulated, the expression of some photosynthesis-related transcripts were down-regulated during the first 24 h, and the expression of some transporter transcripts were up-regulated within the first 2 h. Very interestingly, most transcripts encoding key enzymes involved in flavonoid biosynthesis were highly expressed regardless of starvation, while transcripts encoding laccase, the last rate-limiting enzyme of lignifications, exhibited very low expression abundance in all three samples.

**Conclusion:**

Our study provides a comprehensive expression profiling of *L. punctata* under nutrient starvation, which indicates that nutrient starvation down-regulated the global metabolic status, redirects metabolic flux of fixed CO_2_ into starch synthesis branch resulting in starch accumulation in *L. punctata*.

## Background

Liquid biofuels, such as bioethanol, converted from biomass are considered as a promising alternative for traditional fossil fuels. Biofuels development can reduce greenhouse gas emission and meet the world's rapidly growing demand for energy. Currently, bioethanol is mainly produced from feedstocks with relatively high starch or sugar percentage, such as corn, sugarcane, sweet potato and cassava [1-3]. However, these bioethanol production modes have some inherent problems, including the adverse impacts on food security, environment and insufficient agricultural land [4,5]. Although lignocellulosic sources are also considered as a promising feedstock for bioethanol production, there are several obstacles, such as the lack of an efficient, economical and environment friendly pretreatment process, that still needed to be overcome [6]. Therefore, developing sustainable feedstocks and processing protocols for biofuel production is becoming more and more urgent.

One alternative feedstock is duckweed (*Lemnacecae* family), a small flowering plant that has a global adaptability across a broad range of climates [7,8] and can be easily found in quiescent or slowly flowing and polluted water bodies worldwide [9]. Duckweed has a longer yearly production period than most of other plants, and even grows year-round in some areas with a warm climate [10], which make it a potential sustainable feedstock for industrial application. Previous studies indicated that this plant produces biomass faster than any other flowering plant [11]. With near-exponential growth rates and the shortest doubling times [12-14], duckweed can achieve a biomass of 0.5 to 1.5 metric tons/hectare/day fresh weight or 13 to 38 metric tons/hectare/year dry weight [15]. This accompanies with the tremendous amount of CO_2_ sequestered from the atmosphere and the natural ability of duckweed to thrive on eutrophic wastewater, and recover polluting nutrients [14,16-23], suggesting that growing duckweed for biomass can have large beneficial environmental impacts. In warm seasons, duckweed can remove up to 85% of total Kjehldahl nitrogen (TKN) and 78% of total phosphorous (TP) [19]. Importantly, duckweed biomass exhibits good characteristics for bioethanol production due to its relatively high starch and low lignin percentage [24-27]. High starch accumulation is the most important trait of this crop. Depending on the duckweed species and growing conditions, starch percentage of duckweed ranges from 3% to 75% (Dry weight, DW) [28]. Under nutrient-rich growth conditions, duckweed has a relatively low starch percentage. But by manipulating growing conditions, including the adjustment of pH, phosphate concentration, and nutrient status, starch percentage of duckweed can be significantly increased [26,27,29]. It is encouraging that published studies have demonstrated that carbohydrate derived from duckweed could be converted to ethanol and butanol efficiently [26,27,30-32]. As a novel feedstock for bioenergy production, duckweed has recently gained interest from researchers and governments. In 2009, the United States Department of Energy supported a project to sequence the genome of *Spirodela polyrhiza* (CSP2009, CSP_LOI_793583). Supported by the Minister of Science and Technology, China launched a project to produce liquid biofuel from duckweed biomass, and the first international conference on duckweed application and research was held in Chengdu, China in October, 2011 [8].

Duckweed was once an important model system for plant biology before the days of *Arabidopsis*[33-36] and facilitated important advances in plant biology, such as contributions to our understanding of photoperiod control of flowering [34,37,38], sulfur metabolic pathways and auxin biosynthesis [36]. With the advent of modern genetics and genomics, the status of duckweed as model plant was replaced by other plants, such as *Arabidopsis* and rice, mainly because the exceedingly tiny size and infrequent flowers made genetics studies and breeding in duckweed difficult. With its new value rediscovered, molecular research on duckweed has been carried out again. Several whole chloroplast genome data of duckweed were released [39,40], nuclear genome size were measured by flow cytometry (FCM) [41], and DNA barcoding technology was developed to distinguish different species in *Lemnacecae* family [42-44]. However, as a potential energy crop, functional genomics and transcriptomics data for duckweed are urgently needed.

Next-generation sequencing (NGS) provides a novel method to uncover transcriptomics data [45,46]. This technology shows major advantages in robustness, resolution and inter-lab portability over several microarray platforms [47]. These NGS platforms can detect millions of transcripts and can be used for new gene discovery and expression profiling independent of a reference genome [48-50]. In this study, in order to construct a comprehensive transcriptome and investigate the molecular mechanism behind the starch accumulation in *L. punctata* 0202, samples collected at 0 h, 2 h and 24 time points respectively after fronds were shifted from Hoagland nutrient solution to distilled water were used for a high throughput RNA-Seq analysis. Paired-end (PE) reads from the RNA-Seq were then de novo assembled to build a duckweed transcriptome, which was further used for comparative analysis to reveal the expression patterns of this gene set. This analysis gives a preliminary but global insight into the possible molecular mechanism of starch accumulation, and provides large amount of information for future basic research in duckweed.

## Results

### Effect of nutrient starvation on *L. punctata* growth and starch accumulation

**Figure 1:**
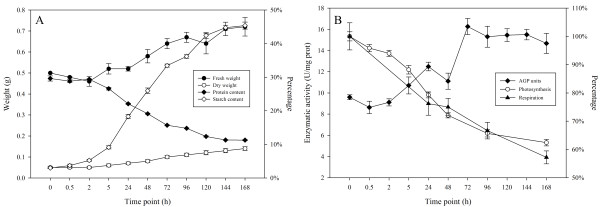
**Component, photosynthesis and respiration of nutrient starvation treated *L. punctata *and activity of ADP-glucose pyrophosphorylase.** Fronds were collected at different time point and used for fresh weight, dry weight, protein and starch percentage analysis, and enzymatic activity assay for ADP-glucose pyrophosphorylase (EC: 2.7.7.27; AGP) respectively. The protein and starch percentage were calculated basing on dry weight. Activities of AGP were measured following the introduction of Nakamura, Y. et al. [52]. Photosynthesis rate and respiration rate measured at 0 h time point were defined as 100%. For each time point, three culture flasks were chose as replicates for these analyses. A: fresh weight and dry weight correspond to left Y-axis, protein content and starch content correspond to right Y-axis. B: AGP activities correspond to left Y-axis, photosynthesis and respiration correspond to right Y-axis.

*L. punctata* was exposed to nutrient starvation by transferring fronds from Hoagland nutrient solution [51] to distilled water. The fronds were sampled at 0 h, 0.5 h, 2 h, 5 h, 24 h, 48 h, 72 h, 96 h, 120 h, 144 h and 168 h time point respectively for the measurement of fresh weight, dry weight, protein and starch percentage (Figure 1). Results demonstrated that the fresh weight of *L. punctata* increased slowly during nutrient starvation, ranging from 0.50 g at 0 h to 0.72 g at 168 h in one culture flask. Meanwhile, dry weight also increased continuously but faster. At 0 h, the dry weight was 0.05 g, but this value doubled by at 72 h and almost tripled at 168 h. Conversely, the protein percentage dropped to 11.3% (DW) from the original 29.6% (DW), while total protein harvested from one culture flask maintained at 0.015 g. Starch percentage increased fast during nutrient starvation. The starch percentage was 3.0% (DW) at 0 h, but tripled at 5 h. At 24 h and 168 h, starch percentage reached 18.3% (DW) and 45.4% (DW) respectively. The total starch weight increased from the initial 1.5 mg to 63.5 mg in one culture flask, which increased by 42 times. The activity of ADP-glucose pyrophosphorylase (EC: 2.7.7.27; AGP), the most important key enzyme involved in starch synthesis, was also measured. The results showed that enzymatic activity of AGP increased from the initial 9.6 units to 14.7 units per mg of total protein by 168 h.

### Sequencing, de novo assembly and functional annotation of *L. punctata* transcriptome

To investigate the transcriptomic response to nutrient starvation in *L. punctata*, samples collected at 0 h, 2 h and 24 h were used for RNA-Seq analysis. We obtained 41,337,098, 38,628,052 and 38,789,556 PE 90 bp reads from the 0 h, 2 h and 24 h sample respectively, corresponding to 10.7 Gbp in total (Table 1). All of these PE reads were pooled together and then de novo assembled by Trinity (v2012-06-08) [46]. Finally we obtained 74,797 contigs with length ≥200 bp. The average length of these contigs was 1,166 bp, the N50 number was 1,928 bp, and the max length was 16,562 bp. There were 31,989 contigs with length ≥1,000 bp and 13,612 contigs with length ≥2,000 bp (Table 1). The assembled transcriptome sequences (≥200 bp) were deposited in NCBI’s Transcriptome Shotgun Assembly (TSA) database under the accession numbers of PRJNA185389.

**Table 1:** **Assembly quality statistics of the *L. punctata *transcriptome**

**Items**	**Characteristics**
PE read number	118,754,706
Contig number	74,797
Contig ≥2000 bp	13,612
Contig ≥1000 bp	31,989
Average length (bp)	1,166
Max length (bp)	16,562
N50 length (bp)	1,928
Coverage	115.2

PE reads from three *L. punctata* samples were pooled together and assembled by using Trinty (v2012-06-08) [46]. Statistics were conducted by common perl scripts.

**Figure 2:**
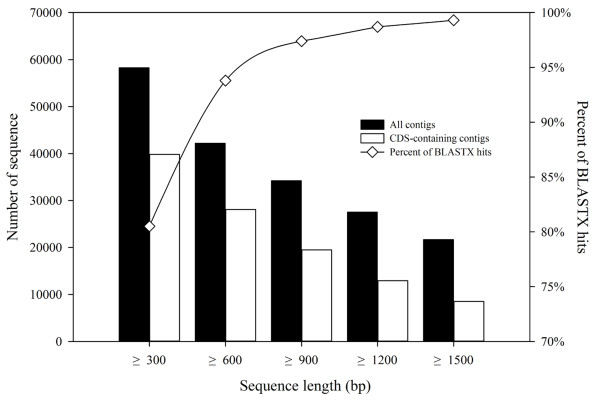
**Annotation rate and long-CDS containing sequences proportion.** 74,797 contigs were used for BLASTX search. The X-axis represents the range of the contig length. Size distributions of the final assembled contigs (black) and number of long-CDS containing contigs (white) shown in vertical histograms correspond to left Y-axis. The percentage of BLASTX hits to size-grouped contigs shown in diamond corresponds to right Y-axis.

To assess the final assembly, we aligned all reads back to the contigs from the assembly using Bowtie2 (v2.0.0-beta5) [53]. As a result, 95.4% of the reads could be aligned back with no more than one mismatch, and the average coverage calculated according to these was 115.2, which demonstrated that almost all reads were utilized for the de novo assembly. Additionally, we calculated the ratios of long-CDS containing transcripts to the total corresponding length contigs. For examples, there were 34,215 contigs with length ≥900 bp, and 19,487 of them (51.6%) contained long-CDS with length ≥900 bp. For contigs with length ≥1,200 bp, this ratio was 45.8% (Figure 2). These results suggested that the final assembly was highly satisfactory.

Sequence similarity search against the Non-redundant protein database (NR) of NCBI [http://www.ncbi.nlm.nih.gov/] was conducted by a locally installed blast program to investigate functional annotation of each contig. Of the 74,797 contigs, 51,968 (69.5%) had significant BLASTX hits and matched 25,581 unique protein accessions (Additional file 1: Table S1). For contigs with length ≥300 bp, 80.5% of them had BLASTX hits, and for contigs with length ≥600 bp, this ratio was 93.8% (Figure 2). These results indicated that most of these contigs were protein-encoding transcripts. GO (Gene Ontology) and KEGG (Kyoto Encyclopedia of Genes and Genomes) annotation were combined with the BLASTX results to give comprehensive functional information for each transcript. In total, we got 105,616 GO annotations for 40,618 transcripts, and 911 unique Enzyme Codes (ECs) number for 8,697 transcripts covering 134 pathways.

### Global molecular characterization of *L. punctata* transcriptome

All PE reads were pooled together for GC percentage statistics by EMBOSS [54] on the Galaxy website [http://main.g2.bx.psu.edu/] [55,56]. It demonstrated that GC percentage of reads is 56.6%, while the transcriptomic GC percentage calculated from the 74,797 contigs is 52.3%. Scanning Open Reading Frames (ORFs) of all contigs, we found there were 40,767 ORFs with length ≥600 bp, and GC percentage of coding regions is 55.2%. For the first position of codon, GC accounts 58.2%. The highest GC percentage presents in the third position, while GC of the second position accounts only 45.0%. We also analyzed the codon usage of these 40,767 ORFs (Additional file 2: Table S2). Results indicated that the most frequently used stop codon in *L. punctata* is TGA, which presents in 55.0% of all ORFs, the second one is TAG (23.9%) and TAA is the least one (21.0%). According to the codon usage analysis, we found that the most abundant amino acids in *L. punctata* are non-polar amino acids (40.5%), and then the uncharged polar amino acids (33.4%), while the acidic and basic amino acids account for 11.7% and 14.5%, respectively (Additional file 2: Table S2).

We used the MIcroSAtellite identification tool (MISA) [http://pgrc.ipk-gatersleben.de/misa/misa.html] to search for simple sequence repeats (SSRs) that were defined as dinucleotide, trinucleotide, tetranucleotide, pentanucleotide and hexanucleotide repeats with at least 18 bp in length. Among 74,797 contigs, a total of 14,250 potential cDNA-derived SSRs (cSSRs) were distributed in 12,707 contigs (Table 2, Additional file 3: Table S3). Among these, 182 are compound cSSRs and 1,415 contigs contain more than one cSSRs. Dinucleotide repeats possessed the highest appearance frequency (70.7%), followed by trinucleotide (25.4%) and tetranucleotide (3.3%) repeats, and only a small portion are pentanucleotide and hexanucleotide repeats (0.3% and 0.3%). Among these cSSRs, the motif AG/CT has the highest frequency (69.2%), followed by motifs CCG/CGG (8.8%), ATC/ATG (5.3%) and AAG/CTT (5.3%).

**Table 2:** **SSR identification of *L. punctata***

**Items**	**Characteristics**
Contig number	74,797
Library size (bp)	87,215,117
SSR number	14,250
SSR density (/Mbp)	163.4
SSR containing contig	12,707
SSR density (/contig)	0.19
Contig containing more than 1 SSR	1,415
Compound formation SSR	182

MISA [http://pgrc.ipk-gatersleben.de/misa/misa.html] was used to search for simple sequence repeats (SSRs) that were defined as dinucleotide, trinucleotide, tetranucleotide, pentanucleotide and hexanucleotide repeats with at least 18 bp in length.

### Differential expression between the samples and qRT-PCR validation

**Figure 3:**
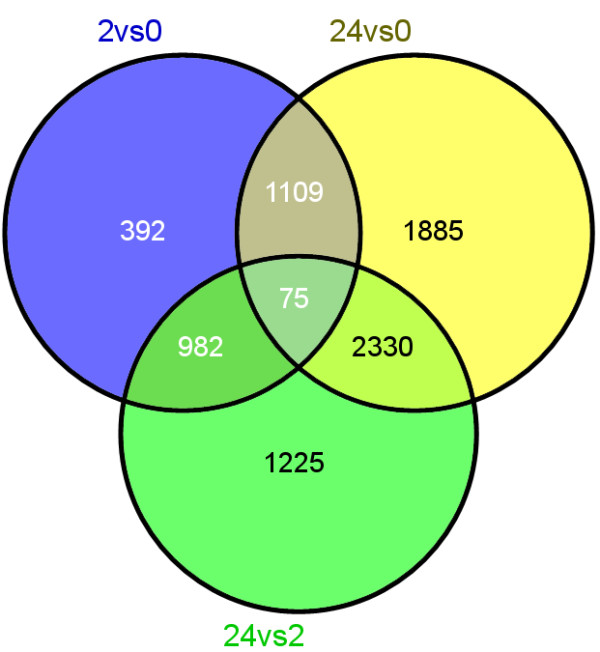
**Differences between each pair of samples.** Overlap examinations were performed basing on the resulting gene lists of three comparisons by VENNY [58]. Overlap among three groups, 2 h *vs* 0 h (blue), 24 h *vs* 0 h (yellow) and 24 h *vs* 2 h (green), were showed here.

To characterize the digital gene expression profiles of *L. punctata*, all PE reads were used for short-read alignment through the perl script provided by Trinity package (v2012-06-08) [46]. For three samples collected at 0 h, 2 h and 24 h, 95.3%, 95.3% and 95.5% of the reads can be aligned back to the 74,797 contigs, and 61.6%, 61.7% and 63.6% were aligned concordantly exactly 1 time, respectively. To balance the effect of library size and the bias introduced by RNA composition, we used edgeR (the Empirical analysis of Digital Gene Expression in R) [57] to make an effective library size for each samples and normalized the number of aligned reads per transcript to FPKM (Fragments Per Kilobase of transcripts per Million mapped fragments) by a RESM-based algorithm. The significant difference analysis was based on the normalized FPKM values. All of the differentially expressed transcripts (DETs) with p-value ≤0.05 and log2 fold-change (log2FC) ≥1 were identified between each pair of samples by edgeR [57]. This generated three DETs sets (2 h *vs* 0 h, 24 h *vs* 2 h and 24 h *vs* 0 h, Additional file 4: Table S4). 2,558 DETs were observed between 0 h and 2 h, 4,612 DETs were found between 2 h and 24 h, while 5,399 DETs were observed between 0 h and 24 h. 75 shared DETs were shared by three comparison sets, 1,184 DETs were shared by comparison set of 2 h *vs* 0 h and 24 h *vs* 0 h, 1,057 DETs were shared by 2 h *vs* 0 h and 24 h *vs* 2 h, and 2,405 DETs were shared by 24 h *vs* 0 h and 24 h *vs* 2 h (Figure 3). All these results showed that the impact on *L. punctata* transcriptome of 2 h nutrient starvation was smaller than that of at 24 h nutrient starvation.

To validate the expression patterns obtained from the comparative RNA-Seq studies, 50 transcripts were selected randomly from the annotated transcripts set for qRT-PCR analysis (Additional file 4: Table S6). The results showed that the expression patterns of 45 of them were consistent with the RNA-Seq analysis, confirming that different expression analysis based on high-throughput RNA sequencing gave reliable expression data in this study.

### Functional classification of DETs

All of the annotated transcripts produced by Trinity and all three DETs sets were grouped into categories based on the GO level 3 annotation and KEGG annotation. Hypergeometric tests were performed with a threshold value of padj ≤0.05 (Additional file 5: Figure S1; Table 3). For GO classification, the annotation information was firstly clustered into three general sections (Molecular Function, Biological Process, and Cellular Component) [59], following an enrichment analysis for each sections separately. As a result, primary metabolic process was the most enriched GO term in the Biological Process sub-ontology. Compared with 0 h time point, 445 DETs and 994 DETs from this category were differentially expressed at 2 h and 24 h respectively. After growing for 24 h under nutrient starvation, the cellular metabolic processes in *L. punctata* were also affected. In the Molecular Function sub-ontology, transferase activity was the most enriched GO term, followed by hydrolase activity. Intracellular part and intracellular organelle were the common enriched elements among these three DETs sets. However, hypergeometric tests based on the KEGG annotation (Additional file 6: Table S5) demonstrated that none of the 137 pathways got significant enrichment. This may because a few key enzymes, rather than most of them, were affected by nutrient starvation.

**Table 3:** Functional enrichment of differentially expressed transcripts among three samples

**Sub-ontology**	**GO terms**	**Reference**	**0 *vs *2**	**pval**	**p.adj**
Biology process	primary metabolic process	11,575	445	0.00011	0.00399
	interspecies interaction between organisms	77	10	0.00022	0.00784
cellular component	intracellular part	12,026	524	5.66E-05	0.00125
	membrane-bounded organelle	9,551	432	0.00017	0.00363
	intracellular organelle	10,868	473	0.00051	0.01122
molecular function	transferase activity	5,774	298	1.91E-09	2.87E-08
					
Sub-ontology	GO terms	Reference	0 *vs* 24	pval	p.adj
Biology process	primary metabolic process	11,575	994	1.71E-09	6.15E-08
	cellular metabolic process	8,913	753	0.00046	0.01653
cellular component	membrane-bounded organelle	9,551	883	8.48E-07	1.87E-05
	intracellular part	12,026	1,037	3.97E-05	0.00087
	intracellular organelle	10,868	947	0.00016	0.00360
	vesicle	1,648	181	0.00171	0.03755
molecular function	transferase activity	5,774	579	1.19E-12	1.79E-11
	hydrolase activity	5,415	490	0.00018	0.0027
	nucleotide binding	5,540	490	0.00092	0.01375
					
Sub-ontology	GO terms	Reference	2 *vs* 24	pval	p.adj
Biology process	primary metabolic process	11,575	866	4.61E-09	1.66E-07
	cellular metabolic process	8,913	649	0.00136	0.04901
cellular component	intracellular part	12,026	889	7.02E-05	0.00154
	intracellular organelle	10,868	805	0.00068	0.01499
molecular function	transferase activity	5,774	506	6.51E-12	9.76E-11
	hydrolase activity	5,415	458	6.68E-08	1.00E-06

GO annotation information of differentially expressed transcripts was clustered into three sub-ontologies, including molecular function, biological process, and cellular component [59]. Then hypergeometric test were performed with threshold value of padj ≤0.05 in each sub-ontology to indentify significant enriched GO terms. Reference means the transcripts annotated to the sub-ontology in the whole transcriptome.

### Expression analysis of photosynthesis-related transcripts

**Figure 4:**
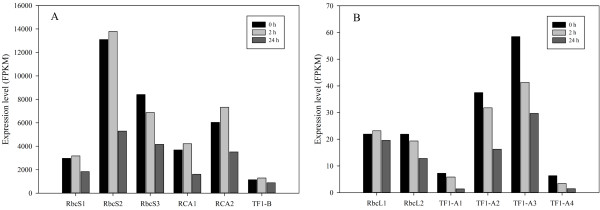
**Expression patterns of some key enzymes involved in photosynthesis and respiration.** RbcS1, RbcS2, RbcS3 were RuBisCO small subunits, corresponding to transcripts comp34400_c0_seq1, comp34400_c0_seq2, comp34400_c0_seq3, respectively. RbcL1 and RbcL2 were RuBisCO large subunits, corresponding to comp17879_c0_seq1 and comp31501_c0_seq6. RCA1 and RCA2 were rubisco activases, corresponding to comp23075_c0_seq1 and comp23075_c0_seq2. TF1-A1, TF1-A2, TF1-A3 and TF1-A4 were ATPase alpha subunits, corresponding to comp26885_c0_seq1, comp26885_c0_seq2, comp26885_c0_seq3 and comp31538_c0_seq1. TF1-B was ATPase beta subunit, corresponding to comp34876_c0_seq1.

Carbon fixed by photosynthesis is the material and energy resource of biosphere and determines the assimilation rate and biomass accumulation [60]. In this study, we found that nutrient starvation decreased the photosynthetic rate (Figure 1). Expression analysis revealed that the expression of transcripts encoding ribulose 1,5-bisphosphate carboxylase/oxygenase (RuBisCO) small subunits (RbcS) and rubisco activase (RCA) declined greatly at 24 h compared to that at 0 h (Figure 4). For example, comp34400_c0_seq1, comp34400_c0_seq2 and comp34400_c0_seq3, three isoforms of RbcS, exhibited expression levels of 2,970 FPKM, 13,105 FPKM and 8,405 FPKM at 0 h, but decreased to 1,837 FPKM, 5,293 FPKM and 4,162 FPKM respectively. Two RCA transcripts, comp23075_c0_seq1 and comp23075_c0_seq2, were expressed at a level of 3,682 FPKM and 6,035 FPKM at 0 h, but 1,606 FPKM and 3,516 FPKM at 24 h. RbcL, which usually is encoded by chloroplast genome, exhibited lower expression level than RbcS in *L. punctata* and did not appear to express differentially under nutrient starvation. Comp17879_c0_seq1 possessed the highest expression levels among several RbcL, with a level of 22 FPKM, 23 FPKM and 20 FPKM at 0 h, 2 h and 24 h, respectively. As RuBisCO is the most critical enzyme in photosynthesis and directly determines the photosynthetic rate, the suppression of RuBisCO and RCA may diminish chloroplast photosynthetic activity. Further analysis demonstrated that the photosynthetic rate of starvation-treated *L. punctata* decreased rapidly. At 24 h and 168 h, this photosynthesis rate dropped to 58.6% and 24.8% respectively compared to that at 0 h. These physiological data matched the expression analysis data described above and suggested that nutrient starvation significantly suppressed the photosynthesis.

### Expression analysis of transporters-encoding transcripts

Expression pattern analysis showed that nutrient starvation resulted in increased expression of most transporters-encoding transcripts, especially some high-affinity transporters-encoding transcripts (Additional file 4: Table S4). Among the 2,558 DETs identified between 0 h and 2 h, 126 DETs encode transporter, including two phosphate transporters, six nitrate transporters, two potassium transporters, six sulfate transporters, five magnesium transporters and three amino acid transporters. For example, two high affinity nitrate transporter-encoding transcripts (comp17450_c0_seq1; comp34106_c0_seq2) had expression levels of 158 FPKM and 93 FPKM at 0 h, but increased to 812 FPKM and 430 FPKM at 2 h. Expression level of a high affinity inorganic phosphate transporter-encoding transcript, comp27235_c1_seq1, was significantly up-regulated from the initial 2 FPKM to 13 FPKM at 2 h. Although most of these transporter-encoding transcripts were up-regulated in 2 h, expression level of two highly-expressed amino acid transporters-encoding transcripts (comp28314_c0_seq3, comp28314_c0_seq4) were down-regulated at least 4 times. Among the 5,399 DETs identified between 0 h and 24 h, 246 transcripts were annotated as transporters. These results suggested that in response to nutrient starvation, *L. punctata* promoted the expression of some ion transporters, especially several high affinity transporters, to adapt itself to extremely low mineral concentrations.

### Expression analysis of transcript encoding key enzymes involved in starch accumulation

**Figure 5:**
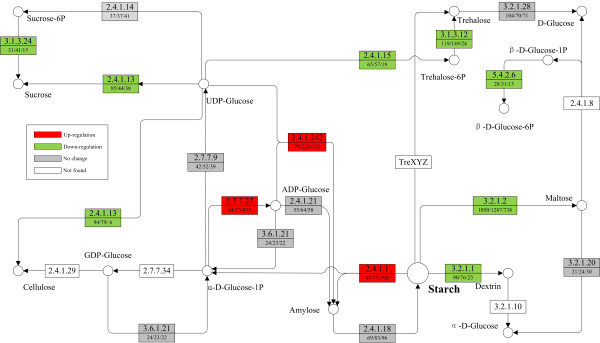
**Expression patterns of some carbon metabolism related transcripts.** Expression variations of some carbon metabolism related transcripts are displayed in the simplified starch and sucrose metabolism pathway. Red boxes indicate the up-regulated enzymes in response to nutrient starvation, green for down-regulated, gray means no significant difference was observed and white means this enzyme was not found in this study. The numbers in the upper half of the boxes correspond to the EC numbers, the numbers in the lower half, separated by slash, correspond to the expression levels of these enzymes shown in FPKM at 0 h, 2 h and 24 h respectively. 2.7.7.27: ADP-glucose pyrophosphorylase; 2.4.1.242: granule bound starch synthase; 2.4.1.1: glycogen phosphorylase; 2.4.1.21: soluble starch synthase; 3.6.1.21: adp-sugar diphosphatase; 2.4.1.18: starch branching enzyme; 3.2.1.1: alpha-amylase; 3.2.1.2: beta-amylase; 2.7.7.9: UDP-glucose pyrophosphorylase; 2.4.1.12: cellulose synthase; 2.4.1.13: sucrose synthase; 2.4.1.14: sucrose phosphate synthase; 3.1.3.24: sucrose-6-phosphate phosphatase; 3.2.1.20: alpha-glucosidase; 2.4.1.15: trehalose-6-phosphate synthase; 3.1.3.12: trehalose 6-phosphate phosphatase; 3.2.1.28: trehalase; 5.4.2.6: beta-phosphoglucomutase.

Starch is the major storage form of sugar and energy in plants. The biosynthesis of this polymer involves several key enzymes, such as soluble starch synthase (EC: 2.4.1.21; SSS), ADP-glucose pyrophosphorylase (EC: 2.7.7.27; AGP), granule bound starch synthase (EC: 2.4.1.242; GBSS) and starch branching enzyme (EC: 2.4.1.18; SBE), while the degradation of starch is usually driven by alpha-amylase (EC: 3.2.1.2) and beta-amylase (EC: 3.2.1.2). To investigate how nutrient stress resulted in starch accumulation, the expression patterns of the transcript encoding key enzymes were analyzed (Figure 5). AGP, the regulator of the first committed step in biosynthesis of starch, is comprised of two identical large subunits (AGP-LS) and two identical small subunits (AGP-SS) in angiosperms, which are encoded by distinct gene families. The AGP-SS is responsible for the catalytic activity while the AGP-LS has a regulatory function. In this study, we identified several transcripts which encode three AGP-LS isoforms and two AGP-SS isoforms. Expression levels of the transcripts encoding three AGP-LS (comp25469_c0_seq1; comp32803_c2_seq6; comp31850_c0_seq1) significantly increased by about 9.0, 7.4 and 6.5 times at 24 h, while the two AGP-SS were slightly up-regulated. Transcript encoding GBSS, a key enzyme in amylose biosynthesis, exhibited an expression level of 79 FPKM at 0 h, and increased to 432 FPKM at 24 h. However, no significant increases were observed for the expression of transcript encoding two other starch biosynthesis regulators, SSS and SBE. We also analyzed the expression pattern of transcripts encoding enzymes involved in starch degradation and some other carbohydrate metabolic branches which compete for substrates, such as alpha-D-Glucose-1P and UDP-Glucose, with starch synthesis pathway (Figure 5). Alpha- and beta-amylase, responsible to the degradation of starch to smaller hydrocarbons, were both down-regulated under nutrient starvation. At 0 h, the highest expressed transcript encoding alpha-amylase had an expression abundance of 90 FPKM, while the highest expressed transcript encoding beta-amylase had an abundance of 1,050 FPKM. At 24 h, the expression level of the highest expressed transcript encoding alpha- and beta-amylase decreased to 23 FPKM and 738 FPKM, respectively. Alpha-D-Glucose-1P and UDP-Glucose, can also be utilized by other pathways for the synthesis of trehalose, sucrose and cellulose (Figure 4). The expression level of transcript encoding trehalose-6-phosphate synthase (EC: 2.4.1.15; TPS) and trehalose-6-phosphate phosphatase (EC: 3.1.3.12; TPP), which catalyze the biosynthesis of trehalose using UDP-Glucose as substrate, were down-regulated by the nutrient starvation. For example, after 24 h nutrient starvation, the expression level of TPP (comp34678_c0_seq1) dropped from 119 FPKM to 24 FPKM. Cellulose synthase (EC: 2.4.1.12; CESAs), sucrose synthase (EC: 2.4.1.13; SuSy) and sucrose-6-phosphate phosphatase (EC: 3.1.3.24; SPP) were also suppressed by nutrient starvation. The findings described here were consistent with the results of enzymatic assay shown above (Figure 1).

### Expression analysis of transcript-encoding key enzymes involved in respiration

Alpha- and beta-amylase are enzymes that can degrade starch to convert it into monosaccharide. Lower expression of these two types of amylase may indirectly decrease the utilization of starch and affect downstream metabolism, such as respiration and some other sugar dependent processes. To investigate whether nutrient stress resulted in a suppression of the starch utilization metabolism, we further studied expression patterns of regulatory enzymes involved in glycolysis and tricarboxylic acid cycle. Interestingly, no significant difference were observed for all of these regulators except the pyruvate kinase (EC: 2.7.1.40; PK; comp17122_c0_seq1) (Additional file 1: Table S1), for which the expression level increased from 53 FPKM to 109 FPKM by 24 h. However, ATP synthase (EC 3.6.3.14; ATPase), which is the energy currency maker that uses the proton gradient generated by the degradation of glucose and electron transfer process to drive the synthesis of ATP, was impacted by nutrient starvation. Usually, ATPase is consisted of two linked multi-subunit complexes, the soluble catalytic core F1, and the membrane-spanning component Fo. The regulatory and catalytic core of F1, alpha and beta subunits, were both down-regulated in *L. punctata* in this study (Figure 4). We identified four alpha subunits and four beta subunits in the transcriptome. All of the four alpha subunits (comp26885_c0_seq1; comp26885_c0_seq2; comp26885_c0_seq3; comp31538_c0_seq1) were down-regulated at least 2 times, while just one beta subunit (comp34876_c0_seq1) was down-regulated. However, the changed beta subunit had much higher expression abundance than the other three. These changes of the energy currency maker may form a bottleneck and affect some ATP dependent metabolic reactions. We also measured the respiration rate of *L. punctata* under nutrient starvation to verify the hypotheses described here. The results showed that the respiration rate of *L. punctata* decreased to 76.6% and 57.2% at 24 h and 168 h respectively compared to that at 0 (Figure 1).

### Lignin and flavonoid biosynthesis of *L. punctata*

**Figure 6:**
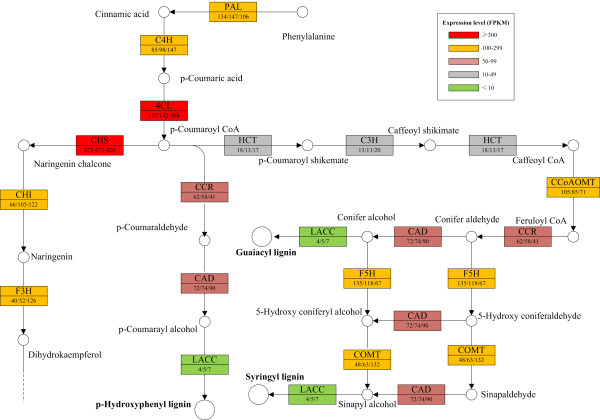
**Expression patterns of lignin and flavonoid biosynthesis related transcripts.** The abbreviations in the upper half of the box correspond to the enzymes involved in lignin and flavonoid biosynthesis, the numbers in the lower half, separated by slash, correspond to the expression levels shown in FPKM at 0 h, 2 h and 24 h respectively. Different colors mean different expression levels. PAL: phenylalanine ammonia-lyase, EC: 4.1. 1.5. C4H: cinnamate 4-hydroxylase, EC: 1.14.13.11. 4CL: 4-hydroxycinnamoyl-CoA ligase, EC: 6.2.1.12. CHS: chalcone synthase, EC: 2.3.1.74. CHI: chalcone isomerase, EC: 5.5.1.6. F3H: flavanone 3-hydroxylase, EC: 1.14.11.9. HCT: hydroxycinnamoyl transferase, EC: 2.3.1.133. C3H: 4-coumarate 3-hydroxylase, EC: 1.14.14.9. CCoAOMT: caffeoyl-CoA O-methyl transferase, EC: 2.1.1.104. COMT: caffeic acid o-methyl transferase, EC: 2.1.1.68. F5H: ferulate 5-hydroxylase, EC:1.14.-.-. CCR: cinnamoyl-CoA reductase, EC: 1.2.1.44; CAD: cinnamyl-alcohol dehydrogenase, EC: 1.1.1.195; LACC: laccase, EC: 1.10. 3.2.

Lignin provides mechanical support for plants to stand upright and enables xylems to withstand the negative pressure generated during water transport, but it is an obstacle for industrial ethanol fermentation from starch as it interferes with the digestion of the carbohydrate fraction of the biomass [61,62]. Flavonoids are secondary metabolic compounds and can exert positive influence on human health and prevent many serious diseases [63,64]. The lignin percentage of duckweed is lower than most of the other plants [23,25,30,65], which makes it an ideal feedstock for biofuel fermentation. In addition, duckweed has high flavonoid percentage in general, which makes duckweed possess the potential to be developed as a source of flavonoids. To study whether low lignin percentage results from low expression levels of lignin biosynthesis related genes, and then leads to the redirection of the metabolic flux into flavonoids just as Besseau *et al*. described in *Arabidopsis thaliana*[66,67], the expression of transcripts encoding for key enzymes for lignin and flavonoid synthesis were analyzed (Figure 6). Phenylalanine ammonia-lyase (EC: 4.3.1.0; PAL), cinnamate 4-hydroxylase (EC: 1.14.13.11; C4H) and 4-hydroxycinnamoyl-CoA ligase (EC: 6.2.1.12; 4CL) are the universal key factors involved in these two routes [68] and exhibited a relative high expression abundances in *L. punctata*. For example, the highest expressed transcript of PAL (comp33246_c0_seq2), C4H (comp17121_c0_seq1) and 4CL (comp35058_c0_seq1) had expression levels of 134 FPKM, 85 FPKM and 157 FPKM at 0 h, and the latter two were slight up-regulated by 24 h. Chalcone synthase (EC: 2.3.1.74; CHS; comp34897_c0_seq1), the pivotal catalyzer for the biosynthesis of flavonoid, showed expression abundance of 362.6 FPKM, 473.3 FPKM and 426.2 FPKM at 0 h, 2 h and 24 h respectively. While the first committed enzymes in lignin biosynthetic branch, cinnamoyl-CoA reductase (EC: 1.2.1.44; CCR), showed a expression level of 62 FPKM at 0 h and 41 FPKM at 24 h. Another key enzyme involved in flavonoid synthesis, chalcone isomerase (EC: 5.5.1.6; CHI; comp17156_c0_seq1), exhibited expression level of 66 FPKM, 105 FPKM and 122 FPKM in three samples. We also identified several transcripts encoding laccases, the last enzyme involved in the last step of lignification. But the expression levels of these laccase-encoding transcripts were extremely low. The highest expressed laccase-encoding transcript exhibited a expression level of only 7 FPKM. The observations described here may be the reasons why duckweed has low lignin percentage.

## Discussion

### Genomics and transcriptomics research of duckweed

Published genomic studies of duckweed mainly focus on chloroplast genome and genome size of different duckweed species [39-41]. However, the sequence of nuclear genome and transcriptome of duckweed is still unrevealed, resulting in the shortage of genetic information, which is still one of the largest obstacles for the development of this crop.

NGS is a cost effective technology to identify millions of transcripts expressed under specific growth conditions and has been widely used since its inception at 2005 [69,70]. In this study, we constructed a comprehensive 87.2 Mbp transcriptome of *L. punctata* by assembling 10.7 Gbp RNA-Seq PE read data*.* As 95.4% of these PE reads can be aligned back to the transcriptome, the corresponding coverage of RNA-Seq PE reads was 115.2. Based on the findings that the genome size of *L. punctata* ranges from 372 to 397 Mbp [41], 10.7 Gbp PE reads used in this study was sufficient enough to cover almost all of the transcripts expressed in *L. punctata*. Using the de novo assembly strategy, a database with 74,797 contigs was established, and 51,968 (69.5%) of them received annotation information from NCBI, KEGG and GO databases. It turned out to be a reliable transcriptome after being assessed by length distribution statistic, map-based and CDS-prediction method. The accuracy of the expression profiling data was verified by real-time quantitative RT-PCR. Furthermore, we identified and characterized 14,250 cSSRs to offer useful molecular markers for future genetic analysis and molecular breeding.

### Transcriptomic changes of *L. punctata* during nutrient starvation

As almost all “essential mineral nutrients” were in deficiency, distilled water provided a global mineral starvation condition which simulates natural water bodies better than the single nutrient-deficient media used in previous published studies [24,71,72]. To cope with the reduced nutrient availability in distilled water, *L. punctata* immediately triggered the up-regulation of the expression of several high-affinity transporters, aiming to increase nutrient acquisition (Additional file 5: Table S4). But this response failed to change the nutrient status and consequently resulted in a series of starvation responses. Under nutritional stress, many aspects of morphology, physiology and metabolism are altered in plants, including adjusting their growth to increase nutrient acquisition [73-78], remobilizing nutrient from inorganic and organic resources [79], and using alternative metabolic pathways that require lower amounts of a limiting nutrient [80,81].

Previous studies have observed that on nitrogen- or phosphate-deficient medium, vegetative growth of duckweed was quickly reduced and eventually ceased. The photosynthesis and respiration rate both fell gradually and roots elongated. At the molecular level, nutrient starvation stimulated starch accumulation while protein or nucleotide percentage decreased [24,26,27,32,72,73]. As “essential mineral nutrients” play crucial roles in cell metabolism and physiology, such as constituents of metabolites and macromolecules, enzyme cofactors, and so on [82], distilled water regulated the metabolic status and induced almost all of the metabolic changes observed previously. For examples, RbcS and RCA of *L. punctata* exhibited very high expression levels in standard Hoagland nutrient solution, but were significantly down-regulated within the first 24 h in distilled water (Additional file 4: Table S4). This observation is consistent with the decrease of photosynthetic rate (Figure 1). The significant down-regulation of the expression level of the alpha and beta subunits of ATPase F1 (Additional file 4: Table S4), together with the decreased respiration rate (Figure 1), reflect the significant suppression of respiration in *L. punctata* under nutrient starvation.

With the shortage of mineral nutrients, especially nitrogen and phosphorus, the biosynthesis of protein and nucleotide in *L. punctata* were restricted and total amount of these two macromolecules were mostly dependent on the initial storage of nitrogen and phosphorus respectively. At the later stage of nutrient starvation, the free nitrogen and phosphorus in *L. punctata* would inevitably decrease due to the majority being fixed in cell skeleton and genomic DNA. This will consequently suppress the total transcription and translation levels. The stable protein content presented in Figure 1 strongly supported this hypothesis. Although the protein percentage decreased from the initial 29.6% (DW) to the final 11.3% (DW) by 168 h, the total protein amount kept at a stable level of 0.015 g per flask. Beside this, deficiency of magnesium, calcium, zinc, iron, manganese and some other minor minerals that play basic functions as enzyme cofactors, widely affected the metabolic reactions in cell, and globally reduced the rate of the metabolisms and energy consumption. The down-regulation of the expression level of the regulatory and catalytic core of ATPase resulted in a decrease of ATP/ADP ratio and then produced a negative feedback regulation of oxidative phosphorylation to decrease the degradation of glucose [83,84]. This indirectly supported the reduction of global metabolism in nutrient starvation treated *L. punctata*.

There are disadvantages in using distilled water as culture media. The obtained data are not enough to explain all the starvation responses since physiological and molecular responses observed in this study might be related to a specific ions deficiency or a mixed stress by the lack of all ions. However, it still provides an alternative method to produce high-starch duckweed. This study provides a preliminary investigation concerning the transcriptomic responses induced by nutrient-stress. Intensive investigations on this topic are needed in the future.

### Starch accumulation of *L. punctata* under nutrient starvation

Although previous studies focused on the starch accumulation of duckweed have made a lot of progresses, no comprehensive assay on this trait has been published and the mechanism of it remains largely unknown. We used a genome-wide transcriptomic analysis method to investigate the expression patterns of some key enzymes involved in starch metabolism.

We integrated the transcriptomics data, the enzymatic assay and starch percentage variation to elucidate the mechanisms of starch accumulation under nutrient starvation. The data from three different levels were analyzed and compared. Starch composition investigation showed that starch percentage of *L. punctata* accumulated rapidly. In the first 24 h of nutrient starvation, starch percentage increased from the original 3.0% (DW) to 18.3% (DW). At 168 h, starch percentage upped to 45.4% (DW) (Figure 1). Calculated according to the dry weight of biomass, the starch net weight increased from 1.5 mg to 63.5 mg per flask, it meant that the starch quantity increased 42 times. Meanwhile, the enzymatic activity of AGP, the regulator of the first committed step in biosynthesis of starch, increased by 30.0% and 52.8% at 24 h and 168 h respectively compared to that of 0 h (Figure 1). More importantly, the expression pattern of key enzyme-encoding transcript involved in starch biosynthesis and degradation further supported these results described above. Findings showed that the expression levels of transcripts encoding AGP-LS were up-regulated significantly, while those of AGP-SS were up-regulated slightly at the first 24 h of starvation, which indicated that the starch accumulation may have originally resulted from the high expression levels of these enzyme-encoding transcripts. On the other hand, nutrient starvation down-regulated expression level of starch degradation related enzymes, such as alpha- and beta-amylase. Additionally, the expression level of regulatory enzymes involved in some competitive bypasses, including trehalose-6-phosphate synthase, sucrose synthase, trehalose 6-phosphate phosphatase, and so on, were also down-regulated (Figure 5). Coulped with the up-regulation of starch biosynthesis related key enzyme-encoding transcripts, these down-regulations finally redirected alpha-D-Glucose-1P and UDP-Glucose to the starch biosynthesis branch.

Taking all of the transcriptomic changes into account, we deduced that the suppression of global metabolic status and cellular respiration decreased the consumption of glucose and redirected the flux toward the biosynthesis of starch. Although several previous studies suggest that starch accumulation is a general response to nutrient deficiency in some plants [85,86], the imbalance between photosynthesis and carbohydrate usage caused by nutrient-deficient is still be less revealed. The findings of this study provides transcriptomics data about this stress response and suggests that the continuous intake of carbon, hydrogen, oxygen and light energy, together with the suppression of many energy-required metabolisms, redirected metabolic flux and resulted in the final starch accumulation.

Transcriptomics data also showed that transcripts encoding laccase, the last key enzyme involved in the biosynthesis of lignin, had a low expression level, while key enzyme-encoding transcripts involved in flavonoid biosynthesis had much higher expression level regaedless of nutrient starvation (Figure 6). This may be the reason that duckweed has low lignin percentage but high flavonoid content. The characteristics of high starch and low lignin percentage, together with high biomass production, make duckweed a promising energy crop.

## Conclusions

This study provides the first comprehensive transcriptome and a genome-wide gene expression profiling of duckweed *L. punctata* exposed to nutrient starvation. Global expression pattern analysis revealed that starvation suppressed most metabolisms due to the extreme shortage of essential mineral nutrients, and then redirected metabolic flux to direct more fixed CO_2_ into starch synthesis pathway, resulting in starch accumulation. These results described here provide valuable genomic resource for duckweed and pave the way for the further molecular biological studies and the application of duckweed as a bioenergy crop.

## Materials and methods

### Duckweed cultivation and growth conditions

*L. punctata* 0202, originally collected from Sichuan, China, was cultivated in standard Hoagland nutrient solution [51] for 14 days under a 16/8 h day/night cycle, with a light intensity of 130 μmol/m^2^/s, and a temperature of 25°C/15°C at day/night. Then 0.5 g fronds were transferred into 50 mL distilled water in 250 mL culture flask for further cultivation over a period of 7 days. A total of 150 culture flasks were used for *L. punctata* cultivation. Eleven different time points, including 0 h, 0.5 h, 2 h, 5 h, 24 h, 48 h, 72 h, 96 h, 120 h, 144 h and 168 h after fronds being transferred into distilled water, were chose for composition and enzymatic activity assay. For each time point, fronds were collected from three culture flasks. Moreover, samples collected at 0 h, 2 h and 24 h were snap-frozen immediately in liquid nitrogen immediately for the further RNA-Seq study.

### Material composition and enzymatic activity assay

For each time point, fronds collected from three culture flasks were dried respectively and used for dry matter, protein and starch percentage measurement. The starch percentage of was measured using a total starch kit (Megazyme, Ireland) according to the manufacturer’s instructions. The crude protein in the biomass was determined as Kjeldahl nitrogen×6.25. Activities of AGP were measured following the introduction of Nakamura, Y. *et al.*[52]. Accordingly, one enzymatic unit of AGP was defined as the amount of enzyme that causes the increase of 0.01 OD at 340 nm of the finally reacted solution per minute. The AGP units were then divided by the total protein amount.

### RNA extraction and library construction

Total RNA were extracted by using OMEGA^TM^ Plant DNA/RNA kit (OMEGA, USA) following the manufacturer’s instruction and genomic DNA were removed by DNase I (Fermentas, USA). The purity, concentration and RNA integrity number (RIN) were measured by SMA3000 and/or Agilent 2100 Bioanalyzer. Qualified total RNAs were then submitted to Beijing Genomics Institute (BGI)-Shenzhen, Shenzhen, China [http://www.genomics.cn] for RNA sequencing.

To get a comprehensive transcriptome and investigate the transcriptomic response to nutrient starvation, fronds harvested at 0 h, 2 h and 24 h were used for RNA-Seq study. More than 20 μg qualified total RNA extracted from each sample were used for RNA sequencing by Hiseq 2000. The poly (A)+ RNAs were purified using poly-T oligo-attached magnetic beads and eluted with Tris-HCl under heating condition. mRNAs was mixed with fragmentation media and then fragmented. Fragmented mRNAs were copied into first strand cDNA using reverse transcriptase and random primers. This is followed by second strand cDNA synthesis using DNA Polymerase I and RNaseH. The ends of these dscDNAs were repaired by adding a single ‘A’ base and then Illumina adapters ligated to the repaired ends. 200 bp cDNAs fragment were purified from a gel and used for further templates enrichment by PCR with two primers that anneal to the ends of the adapters to construct a fragmented cDNAs library. Quality control analysis was performed by Agilent 2100 Bioanalyzer.

### RNA sequencing and de novo assembly

The validated 200 bp fragments cDNAs library constructed above was submitted to Illumina Hiseq 2000 to perform transcriptome sequencing. Then the Illumina sequencing-by-synthesis, image analysis and base-calling procedures were used to obtain PE read sequence information and base-calling quality values.

Sequencing quality were assessed by fastQC [http://www.bioinformatics.bbsrc.ac.uk/projects/fastqc/] and PE reads were de novo assembled by using Trinity (v2012-06-08) [46] under default parameters choice. To investigate the assembly quality, all of the PE reads were aligned back to these contigs by using Bowtie2 (v2.0.0-beta5) program [53], and aligned rate were calculated. Assembly quality was also assessed by length distribution analysis by common perl scripts. N50 number, average length, max length and contig number during different length interval were all been calculated. Moreover, we scanned the best candidate Coding sequence (CDS) for each contig and got the ratios of long-CDS containing transcripts to corresponding length contigs.

### Sequence composition analysis

Before de novo assembly, the GC percentage was calculated basing on the PE reads. After sequence assembly, the GC percentage and codon usage bias of the transcriptome were analyzed using EMBOSS [54] on the Galaxy website [http://main.g2.bx.psu.edu/] [55,56]. We also searched cDNA-derived simple sequence repeats (SSRs) with at least 18 bp in length using a perl script known as MIcroSAtellite identification tool [MISA, http://pgrc.ipk-gatersleben.de/misa/misa.html].

### Functional annotation and cluster

All of the contigs produced by Trinity (v2012-06-08) [46] were used for similarity search against the NR database downloaded from Genebank [http://www.ncbi.nlm.nih.gov/] by using local blast program. For BLASTX search, the threshold was set to E-value <10^3^. The blast results were imported into the Blast2GO [87,88] and performed further functional annotation. Gene ontology (GO) classification [59] was achieved using WEGO [http://wego.genomics.org.cn/cgi-bin/wego/index.pl] [89]. Enzyme codes were extracted and Kyoto Encyclopedia of Genes and Genomes (KEGG) pathways were retrieved from KEGG web server [http://www.genome.jp/kegg/]. KEGG and GO function cluster was conducted by common perl scripts.

### Expression profiling and different expression calling

To investigate the express level of each contig in different samples, all PE reads for each sample were aligned back to the final assembly by using perl scripts in Trinity (v2012-06-08) package [46] under default parameters option. The alignment produced a digital expression levels for each contig and then these were normalized by RESM-based algorithm by using perl scripts in Trinity (v2012-06-08) package [46] so as to get FPKM values.

Based on the expression levels, the effect and bias introduced by library size and/or RNA composition were eliminated by edgeR (the Empirical analysis of Digital Gene Expression in R) [57], then significant DETs among different samples were identified with pvalue ≤0.05 and log2 fold-change (log2FC) ≥1. The cluster of the DETs was performed by using the common perl and R scripts.

### Expression levels verification

To verify the reliability of the NGS-based expression levels, 50 transcripts were chose randomly from the annotated transcripts set for primer design (Additional file 7: Table S6) and real-time quantitative RT-PCR analysis. First strand cDNA was synthesized from 500 ng assessed total RNA using oligo (dT) and random hexamers as primers using Moloney murine leukemia virus (M-MLV) reverse transcriptase (Invitrogen, CA, USA) according to the manufacturer’s instructions. Real-time PCR were performed using Ssofast Evagreen supermix (BIO-RAD, CA, USA) on CFX Connect Real-time PCR detection system (BIO-RAD, CA, USA). The quantitative RT-PCR was implemented under the following program, 5 min at 95°C, followed by 40 cycles of application with 10 s of denaturation at 95°C, 30 s of annealing at 60°C and 30s of extension at 72°C. Three biological replications were used and amplicons were used for meltcurve analysis to check the amplification specificity. The relative expression levels were calculated using Vandesompele method by CFX Manager 2.1 of the amplifier.

## Abbreviations

DW: Dry weight; NGS: Next-generation sequencing; PE: Paired-end; NR: Non-redundant protein database; ORF: Open reading frame; FPKM: Fragments Per Kilobase of transcripts per Million mapped fragments; DET: Differentially expressed transcript; log2FC: log2 fold-change; GO: Gene Ontology; KEGG: Kyoto Encyclopedia of Genes and Genomes; EC: Enzyme codes.

## Competing interests

The authors declare that they have no competing interests.

## Authors’ contributions

XT carried out the data analysis, drafted and revised the manuscript. YF participated in the design of the study and revised the manuscript. YX carried out the biochemical assays, drafted and revised the manuscript. Y-lJ participated in the design of the study and revised the manuscript. X-rM revised the manuscript. YZ revised the manuscript. K-zH participated in the design of the study. HZ conceived the study and revised the manuscript. H-yW participated in the design of the study and revised the manuscript. All authors read and approved the final manuscript.

## Supplementary Material

Additional file 1: Table S1 Sequence annotations of L. punctata transcripts and the gene expression profiling of three samples. Click here for file

Additional file 2: Table S2 Codon usage table of L. punctata coding sequences. Click here for file

Additional file 3: Table S3 Statistics of cDNA-derived SSR markers in L. punctata. Click here for file

Additional file 4: Table S4 Differentially expressed transcripts identified among three L. punctata samples. Click here for file

Additional file 5: Figure S1 GO classification of L. punctata transcriptome and differentially expressed transcripts indentified among three samples. A: GO classification of the L. punctata transcriptome; B,C,D: GO classification of the differentially expressed transcripts for 0 h vs 2 h, 0 h vs 24 h, 2 h vs 24 h. Click here for file

Additional file 6: Table S5 KEGG classification of L. punctata transcriptome and differentially expressed transcripts indentified among three samples. Click here for file

Additional file 7: Table S6 Comparison of expression patterns between RNA-Seq expression and qRT-PCR. Click here for file
