# Supplementary figures and images for: Comparative transcriptome analysis to investigate the high starch accumulation of duckweed (Landoltia punctata) under nutrient starvation
Source: Biotechnol Biofuels. 2013 May 8;6:72. doi: 10.1186/1754-6834-6-72 (PMC3654882; doi:10.1186/1754-6834-6-72)

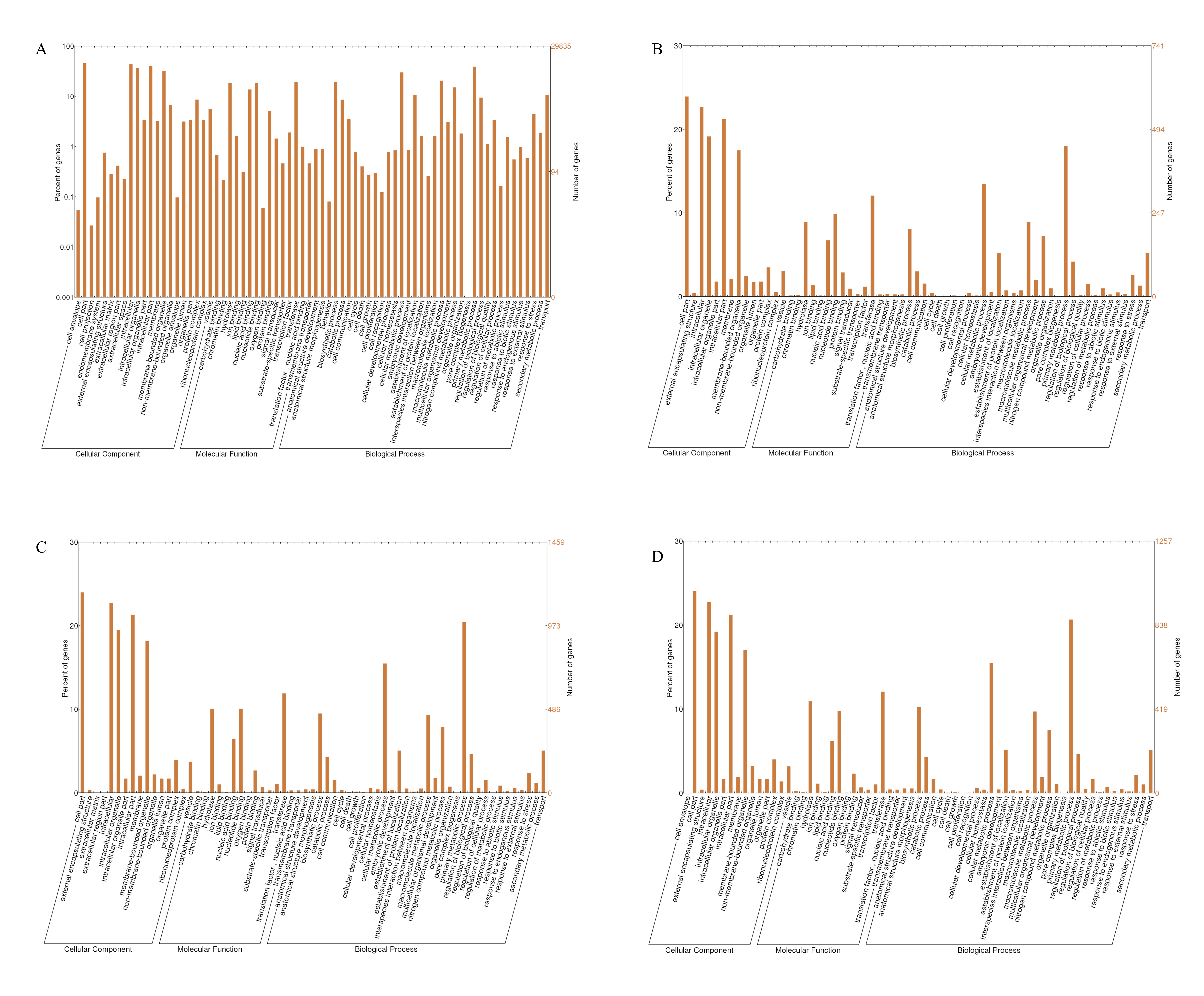

Supplement: Additional file 5: Figure S1 — GO classification of L. punctata transcriptome and differentially expressed transcripts indentified among three samples. A: GO classification of the L. punctata transcriptome; B,C,D: GO classification of the differentially expressed transcripts for 0 h vs 2 h, 0 h vs 24 h, 2 h vs 24 h. [file 1754-6834-6-72-S5.png]
